# Measurement Tools for the Immersive Visualization Environment: Steps Toward the Virtual Laboratory

**DOI:** 10.6028/jres.112.019

**Published:** 2007-10-01

**Authors:** John G. Hagedorn, Joy P. Dunkers, Steven G. Satterfield, Adele P. Peskin, John T. Kelso, Judith E. Terrill

**Affiliations:** National Institute of Standards and Technology, Gaithersburg, MD 20899-8911

**Keywords:** immersive visualization, measurement, tissue engineering, virtual laboratory, virtual reality

## Abstract

This paper describes a set of tools for performing measurements of objects in a virtual reality based immersive visualization environment. These tools enable the use of the immersive environment as an instrument for extracting quantitative information from data representations that hitherto had be used solely for qualitative examination. We provide, within the virtual environment, ways for the user to analyze and interact with the quantitative data generated. We describe results generated by these methods to obtain dimensional descriptors of tissue engineered medical products. We regard this toolbox as our first step in the implementation of a virtual measurement laboratory within an immersive visualization environment.

## 1. Introduction

The National Institute of Standards and Technology (NIST) was established in 1901 as the National Bureau of Standards. Although NIST’s mission has expanded over the years, metrology, or measurement science, has remained a central theme. In recent years, it had become apparent that we could advance measurement science by bringing interactive measurement methods into the immersive environment. Immersive visualization provides a sense of being physically present in the same space with virtual data representations. As we move through the virtual environment and examine these virtual objects, we observe their physical extents, shapes, alignments, and separations. All of these dimensional properties can be measured, so why not use the virtual environment itself as a vehicle for making these measurements?

While it would be ideal to derive measurements in the course of the physical experiments during which the original data are acquired, this is not always possible. For example, tomographic data (such as the tissue engineering data described below) require a reconstruction phase to get it into a form where we can begin to derive measurements of length, area, or volume. This is also true of computational experiments in which data may be generated during computational runs, but those data might not be amenable to direct measurements. Measurement from within the immersive environment provides unique capabilities in this regard. In particular, the interactive nature of the immersive environment allows the researcher to apply scientific judgment in identifying features of interest and performing manual measurements of those features.^1^ When automatic measurement algorithms do not yet exist, the informed judgment of the scientist can be effectively exercised in the virtual environment.

**Fig. 1:**
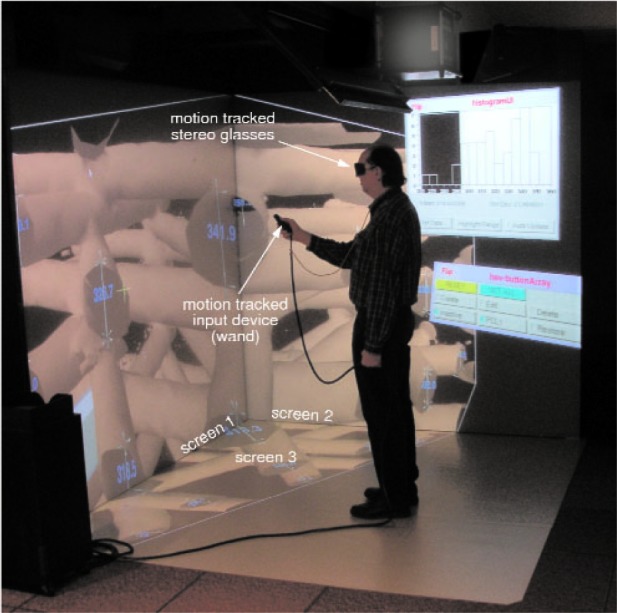
A user in the NIST immersive visualization environment.

The Scientific Applications and Visualization Group at NIST has been collaborating with NIST scientists on a variety of projects involving scientific visualization. As part of those efforts, we have created an immersive visualization system that is pictured in Fig. 1. This figure indicates several important components of the system: the three screens that provide the visual display, the motion tracked stereoscopic glasses, and the handheld motion tracked input device. The screens are large projection video displays that are placed edge-to-edge in a corner configuration. These three screens are used to display a single three-dimensional (3D) stereo scene as shown in the figure. The scene is updated based on the position of the user as determined by the motion tracker. This allows the system to present to the user a 3D virtual world within which the user can move and interact with the virtual objects. The main interaction device is a hand-held three button motion-tracked wand with a joystick.

We have been using this virtual environment for various applications such as visualization of chemical simulations, microscopy, and rheological properties of concrete [1]. In these applications, we have used the immersive visualization system to provide the scientist with views and interactions for a qualitative experience of data. The user can look at data representations at any scale and position, move through data and change scale and orientation easily, and control the elements of the virtual world using a variety of interaction techniques.

This is fairly typical of the current use of virtual reality for scientific visualization. The technology provides the researcher with the ability to perform qualitative tasks with the virtual data representations such as look ing for patterns or seeing spatial relationships among elements. Uses of immersive visualization for applications such as architectural walk-throughs [2] and psychological treatment [3] are clearly using the technology to provide the user with a subjective qualitative experience.

Although we intend this measurement work to be useful in many contexts, our driving application is tissue engineering, which will be described in more detail below. We had been using our immersive visualization system to provide researchers with qualitative views of their tissue engineering data [4], and it was a natural step to begin to use it to extract quantitative information from the virtual scene. With the tools that we describe here, the researcher can make measurements of virtual objects and perform analyses of those measurements within the virtual environment. In effect, we have made the immersive visualization environment into a scientific instrument that gives us the ability to acquire and process data.

It is these tools that are the main subject of this paper. This project is a first step toward a true virtual laboratory [4]. We envision the virtual laboratory as a place where researchers interact with ongoing physical experiments and simulations using interactive tools for visualization, measurement, and analysis of the experimental or simulation data. This first step relies on manual measurement methods based on researchers’ scientific judgements.

This work is not the first attempt to perform measurements in a virtual reality environment or desktop visualization system. For example, Crumbs [5] is a system that allows researchers to manually measure the lengths of fibers in volume data sets. Bethel [6] describes a system that uses a virtual protractor and caliper to measure angles and distances from stereo image pairs generated by a scanning electron microscope. The most pertinent prior work has been done for two medical applications [7, 8]. The tools in these projects measure distance, volume, and angles, and both operate on desktop systems. A *virtual tape measure* [9] has been developed to aid in micro-surgery that operates in an augmented reality environment. The most relevant work is described by Reitinger [10] who describes a set of virtual reality tools for making measurements (distance, angle, and volume) for surgical planning. They point out that the virtual environment affords more natural interactions, so the user is able to make measurements more effectively than could be done on a desktop system. Our work involves the implementation of some similar measurement tools, some novel tools, and incorporates more tools for statistical analyses and investigation of the measurement data derived from the 3D scene. Moreover, the toolbox is not specific to any one application; the tools are general and can be moved from application to application.

## 2. The Tissue Engineering Application

The project that has motivated this work involves the characterization of materials used in tissue engineered medical products (TEMPs). Tissue engineering is an emerging interdisciplinary field that has evolved because of the dire need for compatible replacement organs and tissues in light of the shortages of transplantable organs and the problems associated with biomaterial implants [11].

The term *tissue engineering* is defined as: “The application of principles and methods of engineering and life sciences toward a fundamental understanding of structure-function relationships in normal and pathological mammalian tissues and the development of biological substitutes to restore, maintain, or improve tissue function.” [12] TEMPs often consist of a synthetic or naturally-derived scaffold that provides form and foundation for cells as they produce the tissue of interest. Successful TEMPs will allow cell infiltration and foster proliferation and differentiation within the scaffold.

According to a recent review by Lysaght [13], 20 TEMPs had entered Food and Drug Administration clinical trials by the end of 2002. Four were approved, and none are commercially successful despite sound technology behind the products. Some of the reasons for the lack of commercial success cited in this review are: improvement over existing therapies either are not large enough or are for too small a patient group, protracted and costly regulatory approval caused the products to require a very large return on investment, ineffective marketing, limited physician and user acceptance, and lack of a low cost manufacturing approach. Tissue engineering and regenerative medicine have been identified by NIST as biotechnology areas where metrology development is required to lower the cost barrier for product commercialization. To this end, the Biomaterials Group at NIST is developing measurements and methods to quantify cell/scaffold interactions. In this case, we are quantifying how the structure of these scaffolds influences cell response and nutrient and waste transport. We are quantifying structural descriptors such as porosity, pore size distribution, pore connectivity and tortuosity, strut size distribution, strut planarity and orientation, and anisotropy measures of the aforementioned descriptors. Using the immersive visualization environment lets us simultaneously spatially encode the values of these descriptors within a 3D image of the scaffold and see correlations not readily observable with desktop displays.

Some techniques traditionally used to quantify scaffold structure include scanning electron microscopy, mercury and flow porosimetry, gas adsorption, and pycnometry [14]. These techniques probe a limited number of descriptors of interest—most commonly porosity, pore size distribution, tortuosity, surface area, permeability, and compressibility—with caveats. The biggest drawback to all of these techniques is that they do not provide a direct measure of scaffold structure in three dimensions. X-ray micro-computed tomography (X-ray μ-CAT) provides a direct measure of scaffold structure and has been used to generate 3D images of scaffolds [15, 16]. There have been several efforts to quantify the results from using this technique for both scaffold structure [14, 17] and mineralization within bone tissue constructs [18, 19].

**Fig. 2:**
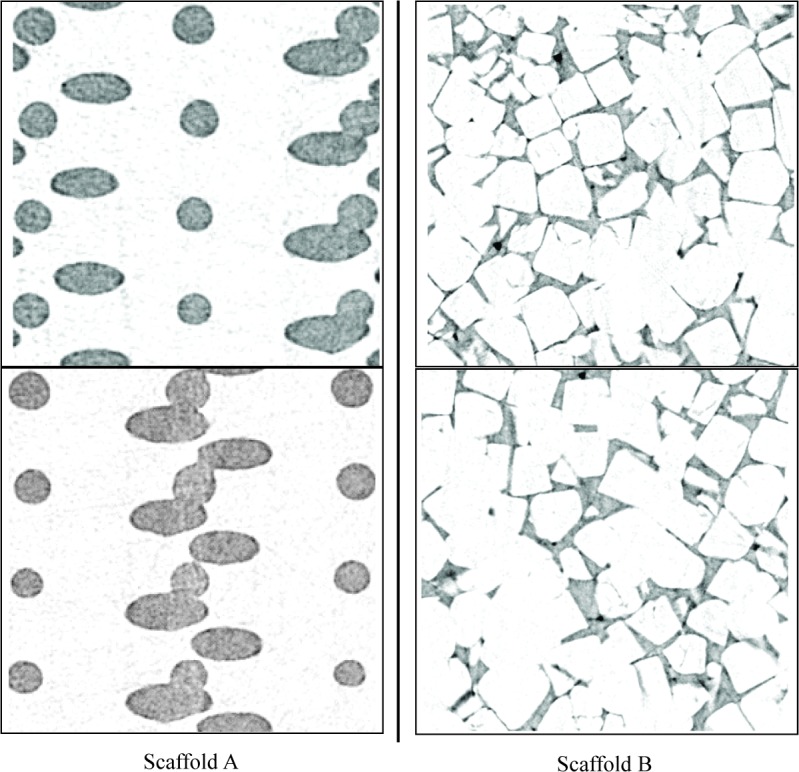
Two-dimensional slices of X-ray μ-CAT data for both Scaffold A and Scaffold B.

**Fig. 3:**
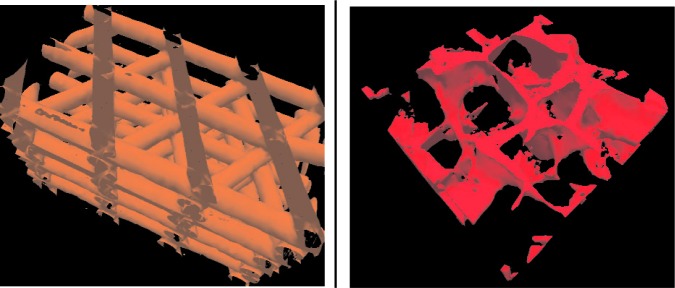
Three-dimensional representations of the X-ray μ-CAT data. for Scaffolds A and B.

In this work, we use X-ray μ-CAT images from two types of structures commonly used in orthopedic applications: regular structures manufactured using rapid prototyping (RP) methods (Scaffold A) and random structures fabricated with salt-leaching (Scaffold B). Figure 2 shows two-dimensional (2D) X-ray μ-CAT images of Scaffolds A and B, and Fig. 3 shows 3D representations of both Scaffolds A and B. The characteristics of these two scaffold types will be described in more detail in Sec. 5.

Scaffold A is being considered as a geometric reference scaffold. A geometric reference scaffold has a well-defined and reproducible geometry to control the effect that geometry has on cell response so that other factors such as cell type or growth factors can be investigated. We are interested in how well Scaffold A adheres to its design specifications and in fabrication variability. The scaffold descriptors of interest are: fiber diameter and aspect ratio, fiber orientation, parallelism of fibers, planarity of fiber layers, and angles between fibers. Cell ingrowth is an issue for structures like those of Scaffold B. Our intent is to develop tools that measure not only the size of the pores but also the size and number of connections between pores. This information is critical in designing scaffolds that enable cell ingrowth and efficient transport of nutrients and waste.

## 3. The Tools

Clearly, it would be desirable to develop automated methods or a hybrid mixture of automated and interactive methods for measuring these scaffold descriptors, but such methods do not yet exist. In their absence, we have pursued purely interactive methods implemented within the immersive visualization environment. We designed a collection of general-purpose tools to address the immediate research needs of the tissue engineering project. Our objective was to build a software system within the virtual environment that integrates measurement, analysis, and interactions that link analysis results with the visualization. More specifically, we envisioned the following scenario:

- The user manually collects a series of measurements.
- A statistical analysis is made.
- This analysis, including a representation of the distribution of measurements, is presented to the user in the virtual environment as either a table or a histogram.
- The user can then interact with the display of the measurement distribution in order to highlight measurements (in the virtual scene) that fall within any selected range of values.

All of these tasks should be performed in real-time during the immersive visualization session. We designed and implemented the following tools to support the measurements needed for the tissue engineering project:

- LineMeasure
- CylinderMeasure
- EllipsoidMeasure
- WandClip
- BoxClip

The first three tools produce measurements: the other two tools are supporting software intended to improve the utility of the measurement tools. Each of the three measurement tools places objects into the virtual world that are, in some sense, surrogates for the features being measured. The researcher can position and stretch those surrogate objects to fit the features of the data representations under study. The measurement of those features can then be taken from the known dimensions of the surrogates, and the user is able to analyze and interact with those measurements.

We envision these tools as the first of a set that will form a general purpose tool-box for the measurement of dimensional quantities in a virtual laboratory.

### 3.1 LineMeasure

**Fig. 4:**
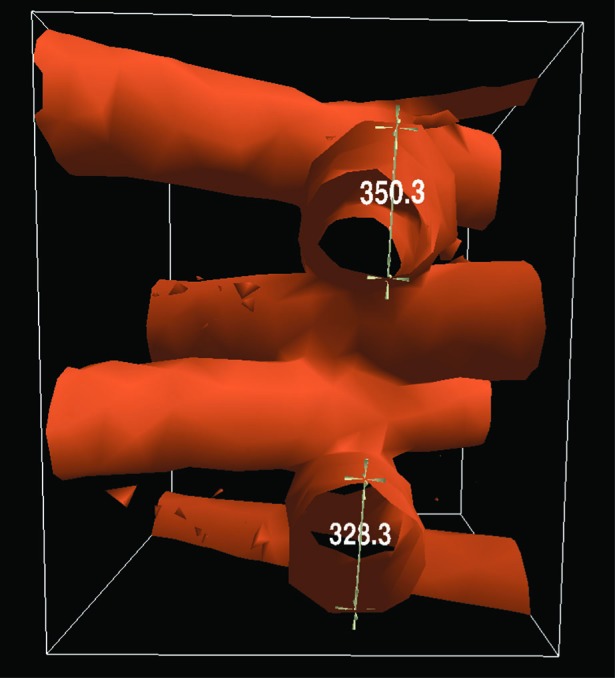
The LineMeasure tool. Two measurements are shown measuring the diameters of two struts of a Scaffold A sample.

With the LineMeasure tool, the user can measure the linear distances between pairs of points in the virtual scene. Each measurement is represented visually by a line connecting the points, 3D markers at each end point, and a text display of the length of the line segment. See Fig. 4 for an example showing two such measurements. The interface is modal with four modes: create mode, edit mode, delete mode, and inactive mode. The user switches modes by making selections using the menuing system.

In create mode, the user can make a new measurement by moving a 3D cursor to the desired location and pressing a wand button dedicated to this tool. After the first point is selected, a 3D rubberband line is displayed between the first anchor point and the current location of the wand. A numeric text display is superimposed over the line. The text is continually updated to show the current length of the line. The user moves the wand to the second point and presses the wand button again to complete the measurement.

In edit mode, the user can modify existing measurements, and in delete mode the user can remove measurements. The user can also go into inactive mode which simply causes the tool to ignore all user input except for selecting one of the other modes; this mode is useful during scene navigation and measurement inspection.

**Fig. 5:**
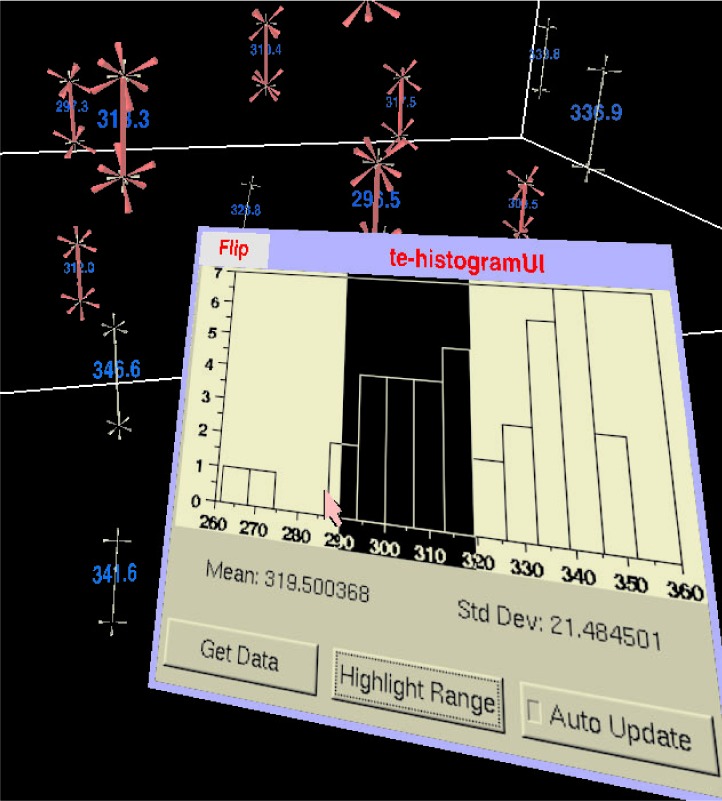
The analysis panel for the LineMeasure tool as it might appear in the 3D scene. The user has swept out a range of lengths from about 290 to 320 on the histogram and the corresponding measurements have been highlighted in the virtual world.

At any step in the process, the user can analyze and interact with the current set of measurements through a panel that is displayed by the menuing system within the virtual scene. Figure 5 shows the panel in use in the context of the virtual scene. When the user presses the *Get Data* button on the panel, a histogram of the line segment lengths together with their mean and standard deviation are displayed. The user can use the wand to sweep out a portion of the histogram and have the system highlight the corresponding line segment measurements whose lengths lie in that portion of the histogram.

Note that the use of the rubberband line is a standard technique in desktop interfaces for interactively connecting two points. We felt that users would find this a familiar (and thus easy to use) feedback mechanism. The numeric display of the line length while it is being stretched to the desired points was intended to provide the type of quantitative information that one might see on a technical drawing, with the added benefit of continual update during the interactive operation. Note also that the highlighting based on interaction with the histogram could be regarded as a form of an interactive technique known as *brushing* [20].

### 3.2 CylinderMeasure

**Fig. 6:**
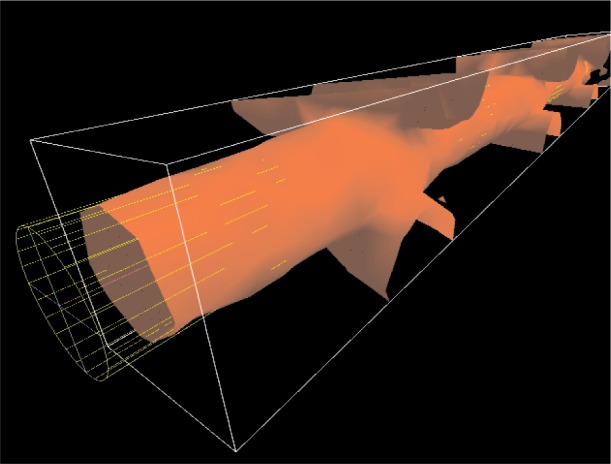
The CylinderMeasure tool in use. The user has fit the cylinder surrogate object to a strut of the Scaffold A sample. Note that this strut has been isolated by use of the BoxClip tool (see Sec. 3.5).

This tool enables the user to interactively measure tube-like structures. This is done by placing a 3D wire-frame representation of a quasi-cylinder unto the scene and moving that object to approximate the position of the tube structure. This quasi-cylinder (which we will refer to simply as a cylinder) is a tube with elliptical cross section that can be manipulated to change its length, either cross sectional axis, and orientation. Figure 6 shows the CylinderMeasure tool in use.

The interface of CylinderMeasure is also modal. In this case, there are three modes: create/edit mode, delete mode, and inactive mode. For this tool, we combine create and edit modes to minimize the number of times that the user must change modes.

In create/edit mode, the user can create a new cylinder measurement or modify an existing measurement. Again, the primary interaction is through the motion-tracked wand to which a virtual 3D cursor is attached. When the cursor is not in proximity of an existing cylinder measurement, pressing the wand button causes the creation of a new cylinder measurement placed at the location of the 3D cursor with a standard size and orientation.

The user can move any cylinder measurement object by grabbing it by its center and dragging, while the length and orientation of the cylinder can be changed by grabbing and dragging end-points of the cylinder. The lengths of the cross sectional axes can be interactively changed by dragging axes end points; this action can also be used to reorient the elliptical cross sections rotationally about the longitudinal axis.

Similar to the LineMeasure tool, there is a delete mode in which the user can delete existing cylinder measurements, and an inactive mode that causes the tool to ignore user input except for moving to another mode.

Interactive analysis of these cylinder measurements can take several forms. We have implemented several analyses that can be accessed during the immersive session:

- average diameter
- longitudinal direction
- aspect ratio of cylinder cross section
- eccentricity of cylinder cross section.

Except for longitudinal direction, each of these analyses present a mean and a standard deviation, together with a histogram in a panel in the virtual scene; the display is similar to that in Fig. 5. The longitudinal direction analysis is done by calculating an average 3D direction for the longitudinal axis of the cylinder using spherical weighted averaging [21]; angular deviations of each measurement are then calculated for each measurement and a histogram of these deviations is presented to the user in the virtual scene. In all cases, the user can interactively sweep out a range of the histogram and highlight the measurements in that range in the 3D scene.

It should be noted that there are other sorts of analyses that could be done for the cylinder measurements. For example, we could provide statistics on the volume, length, or cross sectional area of the cylinders.

### 3.3 EllipsoidMeasure

**Fig. 7:**
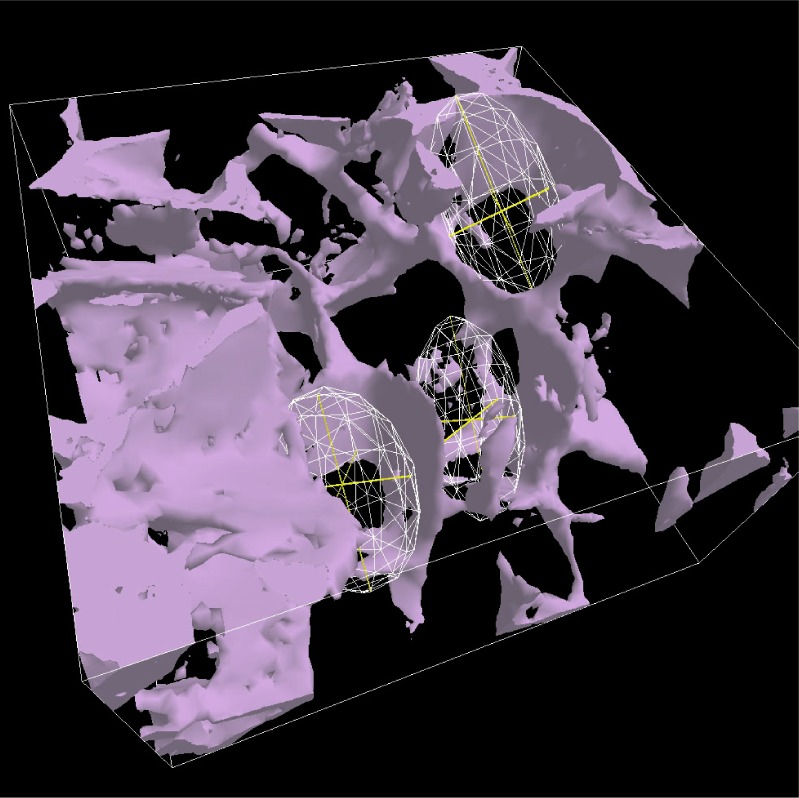
The EllipsoidMeasure tool in use to measure pore size in the Scaffold B material.

The EllipsoidMeasure tool gives the user the ability to measure 3D regions that are ellipsoidal in nature. The user places a sphere into the 3D scene, then stretches, drags, and orients it to form an ellipsoid that fits the region to be measured. Each of the three axes can be independently changed and the location and orientation of the ellipsoid can be directly manipulated. Figure 7 shows the tool in use.

As with the previous tools, the interface is modal. In create/edit mode, the user can place a new measurement with a point and click using the motion tracked wand. The initial ellipsoid is a wire-frame sphere with X, Y, and Z axes displayed to the user. The user can pick any of the six axis end-points to stretch or compress each dimension or to reorient the ellipsoid; the center is fixed during axis end-point manipulations. The user can also select the center point of the ellipsoid to drag it to a new position.

Again, the tool has both delete and inactive modes that operate as in the CylinderMeasure and LineMeasure tools.

As for the CylinderMeasure tool, there are many analyses of the ellipsoid data that we could have provided to the user during the interactive session. For our initial purposes, we implemented the following analyses:

- 3D direction of the longest axis
- length of the longest axis
- eccentricity of the cross section perpendicular to the longest axis

The length and eccentricity measurements are presented with the mean, standard deviation and histogram display in a panel similar to that in Fig. 5. The 3D direction of the longest axis is handled much as the 3D direction of the longitudinal axis of the cylinder measurements as described above. The interaction with the histogram and the highlighting behavior is provided in a similar way.

### 3.4 WandClip

The WandClip tool lets the user discard portions of the 3D scene to reveal interior structure that may be hidden. When this tool is activated. a virtual plane (indicated by a wireframe rectangle) is attached to the 3D position of the wand. The position of this plane relative to the wand is configurable, but is usually specified to be about 0.2 m away from the wand in real space and oriented orthogonal to the Y axis of the wand. The tool is configured such that it can act on some elements of the virtual scene while leaving others unaffected.

**Fig. 8:**
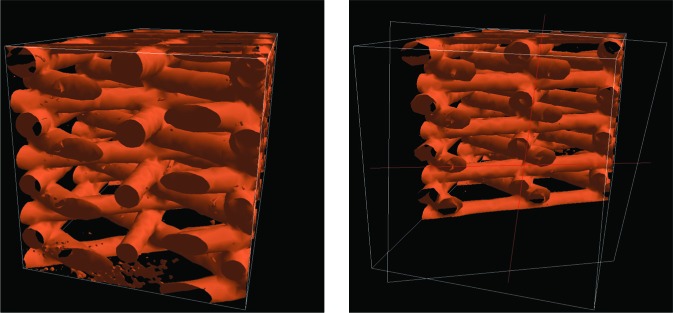
The WandClip tool in action. The image on the left shows a complete Scaffold A data set. The image on the right shows the samce data set with the interior revealed by the WandClip tool.

As the user moves the wand in the 3D scene, the plane cuts into the scene, acting like a sort of window into the interior structure of objects. It is as if the user is controlling the position of a sheet of glass and everything that is closer to the viewer than that sheet is removed from the scene. Figure 8 shows the operation of WandClip. This tool is not directly tied to the operation of the measurement tools, but it is intended to help the user to make preliminary identification of structures to be measured.

### 3.5 BoxClip

The BoxClip tool is related to the WandClip tool in that it clips out portions of the 3D scene in order to reveal hidden features. However the BoxClip tool is not always tied to the position of the wand; the user can position a clipping box that remains spatially stable in the virtual scene.

**Fig. 9:**
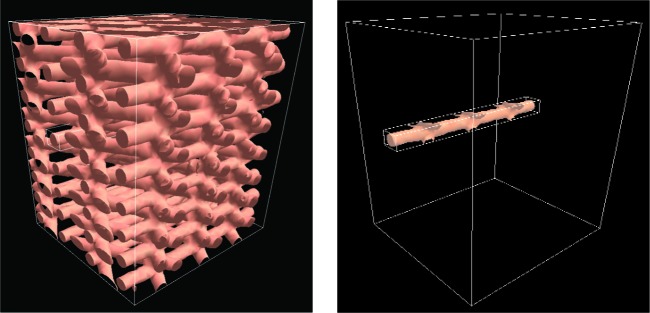
The BoxClip tool used to reveal a single strut of Scaffold A material. The image on the left shows a complete scaffold A data set and the image on the right shows the action of the BoxClip tool.

The tool presents the user with a wire-frame box. Objects outside of that box are clipped out of the 3D scene. Each face of the box can be selected and dragged with the motion-tracked wand; it is a rubberband box that can easily be stretched and compressed to encompass the desired region. As with WandClip, this tool can be configured so that it will clip some elements and not others. Figure 9 shows BoxClip in action.

Again, this tool is not inherently tied to any of the measurement tools, however we intend to use this tool to facilitate the measurement interactions. The tool can be used to isolate regions of interest and features to be measured. Because the displayed sub-region is spatially stable, measurements can be easily made on the exposed structures.

## 4. Implementation

The underlying software on which our immersive system is built is DIVERSE [22], which provides a portable, modular, open source software platform that manages all aspects of the virtual environment. This includes handling the interfaces to devices (such as motion trackers and user interface devices), stereo parallax, asynchronous viewing frusta, and other functions required for a fully immersive virtual reality system.

There are several features of DIVERSE that are particularly useful in the work described here. DIVERSE provides:

- a flexible scheme for use of the graphics scenegraph
- support for addition of user-supplied components
- support for communication via shared memory between components

The graphics scenegraph is a data structure that describes a hierarchy of relationships among all of the items to be displayed in the virtual scene. DIVERSE provides a structure into which we place the data representations, such as the models of the scaffold material as well as the surrogate objects created by the measurement tools. The scenegraph gives us simple mechanisms for controlling, for example, which items are affected by the clipping tools and which are not.

DIVERSE’s support for user-supplied software components and shared memory communication is central to the way that we build our tools. We add software components to the system through dynamically shared objects (DSOs), which DIVERSE loads at run-time and executes during immersive sessions. DIVERSE executes the code in DSOs in clearly defined and easily controlled ways, and each DSO component can communicate to other DSO components or to external programs via DIVERSE-supplied shared memory tools. Moreover, this shared memory can be networked, allowing these external programs to be executed on any system accessible to our immersive system.

The design and implementation of our measurement tools are inspired, in part. by the *Unix Philosophy* [23] which calls for simple components connected by simple interfaces. In standard Unix programming, this often means small programs, each of which does one thing well, connected by text files (or pipes). In that tradition, we try to design DSOs, each with a limited general-purpose functionality, that communicate through shared memory. Furthermore, non-DSO computation can communicate with DSO components through shared memory, further enhancing our ability to extend the system. Using these mechanisms, we are able to augment the functionality of the virtual environment in an incremental fashion and to build and to combine sets of components that are useful for a variety of applications.

Within this context, we use VEWL (the Virtual Environment Windowing Library) [24] to provide many aspects of our user interface. VEWL is a software subsystem that operates within the DIVERSE framework, with both DSO and non-DSO components. It enables the use of standard desktop user interfaces within the immersive environment. With VEWL, we are able to use simple existing graphical user interface (GUI) tools, such as FLTK (the Fast Light Toolkit) [25], to quickly construct effective user interfaces that operate within the immersive environment. Communication with other DSOs and other external programs are easily accomplished through shared memory.

Each of the measurement tools described above was implemented as a separate DSO. User input such as mode changes, wand button presses, and wand position are conveyed to the DSOs through shared memory. The execution of external programs is also managed through these mechanisms. For example, when the user presses a button on one of the VEWL-menus, an external program, Dataplot [26], is executed to generate statistics and to produce the histograms described above. Dataplot is simply an existing application program that is executed, unmodified, during the immersive session. Information is passed between Dataplot and the DIVERSE DSOs via files.

Another aspect of our implementation approach was to try to provide user interface methods that would be familiar to the user whenever possible. This is why, even with the 3D virtual scene, we use standard 2D desktop GUI mechanisms, as provided by FLTK and VEWL. Another example is our use of the rubberband line in the LineMeasure tool; this is a mechanism that the user is likely to have seen and used in 2D drawing applications.

## 5. Methods

As mentioned above, we use X-ray μ-CAT images of our two scaffold types: the geometrically regular Scaffold A and the randomly structured Scaffold B. The particular RP technology used for Scaffold A is called fused deposition modeling (FDM) [27]. In short, FDM creates successive cross sections of a 3D object. FDM heats the polymer, in our case poly(*ε*-caprolactone) (PCL), to a high temperature to reduce its viscosity and then extrudes the PCL through a small nozzle. As the nozzle moves along both the *x*- and *y*-axis across a foundation, polymer is deposited in a pre-defined pattern and fused with the layer below. Successive cross sectional patterns are laid down until the object is completed. The struts are designed to be 400 μm in diameter and are laid down in a 0-60-120 degree layer pattern. The gap width is designed to be 800 μm.

The salt leached scaffold, Scaffold B, was made by first infiltrating packed sodium chloride crystals with a dimethacrylate resin. The sizes of the sodium chloride crystals were selected by sieving through openings of 250 μm to 150 μm. The samples were photocured, postcured in a vacuum oven, and soaked in deionized water to remove the salt. Details about this procedure are provided elsewhere [28].

The X-ray μ-CAT images were generated with a Skyscan 1072 scanner with voxel spacing of 12.88 μm in each direction. The images were output as a series of bitmap image files. Each 2D image series was assembled into a 3D data set which was then converted to a polygonal surface representation with a combination of custom software and the Visualization Toolkit (VTK) library [29]. These polygonal models were the virtual data representations that were displayed, manipulated, and measured in the immersive visualization environment. Figure 3 shows renderings of the polygonal models for Scaffolds A and B. We generated data for two samples of Scaffold A, which we will refer to as PCL1 and PCL2, and one sample of Scaffold B, the salt leached structure.

In addition to these experimentally derived data sets, we also constructed a virtual model that conforms to the design specifications for Scaffold A as described above; we refer to this as the Scaffold A Synthetic Model. The Synthetic Model is, however, polygonally based so it is an approximation of the ideal structure.

The first of the measurement tools that we implemented was the LineMeasure tool. We used this to measure the diameters of the struts in the PCL1 scaffold. Each strut was measured in several places for a total of 82 linear measurements over the entire sample. Upon completion of the implementation of the CylinderMeasure tool, we manually fit cylinders to each strut in the PCL1, PCL2, and Synthetic Model structures. We used these to measure horizontal gap width, vertical gap width, strut aspect ratio, strut parallelism, and strut planarity. A total of 21 struts were measured in PCL1, 25 struts in PCL2, and 26 struts in the Synthetic Model. We then used the Ellipsoid-Measure tool to try to measure pore size in the salt leached material (Scaffold B); our difficulties in making these measurements will be described in Secs. 6 and 7.

## 6. Results

As the main subject of this paper is the tools themselves, we present only a few of the quantitative results that we obtained from the measurements of Scaffold A to give an indication of the utility of our tools.

Table 1 shows the mean vertical distances between struts (in μm) for the three Scaffold A samples. The results for the Synthetic Model conform well to the design specifications and give us confidence in our measurement methods. The results for PCL1 and PCL2 allow us to compare the design specifications to the manufactured material; the design calls for a center-to-center distance of 1200 μm and an edge-to-edge gap of 800 μm. The statistics show us that these distances are far smaller than expected. Similarly, Table 2 shows the mean angles between adjacent struts for the Synthetic Model, PCL1, and PCL2. In this case, we see very good agreement between the angles specified in the design (60 degrees) and the angles measured by our system for the Synthetic Model as well as the manufactured samples. We should note that for the statistics presented in Tables 1 and 2, the breakdown by orientation was done after the interactive measurement sessions by analyzing data files stored during the sessions.

In addition to these data, we also obtained useful measurements of strut diameter, aspect ratio, planarity, and parallelism for Scaffold A sample. However the measurement of pore sizes in Scaffold B (the salt leached scaffold) using the EllipsoidMeasure tool proved problematic. We were unable to get satisfactory results in this task, which will be discussed more in the next section.

We will use these data to relate dimensional characteristics to functional characteristics of the scaffold materials. Establishing these relationships will require experiments that correlate scaffold structural descriptors with cell response (i.e., proliferation and differentiation).

## 7. Discussion

The measurements made of the Scaffold A Synthetic Model and the derived statistics show very good agreement with the design specification that was used to construct the model. This gives us considerable confidence in the measurements and statistics for the manufactured models (PCL1 and PCL2). It seems likely that the largest source of the error that we see in the Synthetic Model data is due to the difficulty of precisely positioning the tools by hand in the virtual world and to the fact that the Synthetic Model is approximated in the virtual world by a polygonal model.

We can expect that errors of similar or greater magnitude are present in the measurements of PCL1 and PCL2, where the correctness of the polygonal data representations must be assessed. Of course, the assumption that the data representations are true to the underlying physical phenomena is inherent in all use of visualization (immersive and otherwise). The value of any insight gained from a visualization is entirely dependent on the fidelity of the visual representation to the phenomenon being studied. When making measurements in the virtual environment, it will be important to estimate the errors introduced by the use of that technology so that these errors can be included in a statement of uncertainty for the measurements. For example, the accuracy and calibration of the motion tracking system [30] affects the accuracy of the measurements. The assessment of errors specifically attributable to the use of the immersive visualization environment is the subject of a separate project that is in progress at NIST and will not be addressed here. Measurement errors will differ from application to application and from one immersive system to another. It is unclear whether the magnitude of the errors in the measurements described here materially affect our understanding of the scaffold materials.

While using these tools, it became very clear that the scientific judgment of the researchers played a critical role in the accuracy of the results. For example, in using the CylinderMeasure tool, the user must judge where to place the walls of the modeled cylinder, which inevitably must deviate from the walls of the strut as represented in the virtual environment. The results are only as good as the researcher’s judgment. But, of course, the whole point is that the struts are not perfect cylinders and some best-fit must be found. Our tools provide a manual method, but this certainly suggests that we might want to pursue automated methods as well as hybrids of automated and manual methods.

As mentioned above, we were unable to make useful measurements of pore sizes in the salt leached scaffold material using the EllipsoidMeasure tool. We found that the manipulation of the ellipsoid models was reasonably easy and placing them relative to the salt leached scaffold model was not difficult. The problem arose from the nature of the salt leached scaffold data and the polygonal representation generated from those data. We found that the irregular structure of the scaffold material made it difficult for the researcher to unambiguously identify specific pores as well as pore connections. It was felt that any measurements that we made with the ellipsoids would be unreliable.

Concerning ease-of-use, we found that use of the LineMeasure tool was easy and fast. We were able to make many measurements and we have confidence in the statistics generated from these measurements. The use of the CylinderMeasure tool was quite time-consuming, however we felt that the quality of the results were good. Certainly the fact that the angular measurements so closely matched the design specifications serves to verify both the accuracy of the manufacturing process as well as the accuracy of the measurement method. We felt that we were well-compensated for the investment in time in using the Cylinder-Measure tool. The information provided by this tool was very rich and we were easily able to derive many descriptors from the same set of fitted cylinders.

We also found that these manual methods (particularly CylinderMeasure) were tiring for the user. (Note that for our studies, we generally want to make many measurements and calculate statistics. This means that many repetitions of the use of the tool were required.) The fatigue was partly due to the fact that it was difficult to manually drag the surrogate objects to exactly the point desired. The steadiness of the hand became a factor and many small adjustments were often required to get the desired result. This problem could be somewhat mitigated by enlarging the scale of the virtual scene so that small mis-positionings would have less impact. This rescaling, however, presented its own difficulties when navigating through the larger virtual world.

Another important user-interface issue was visual clutter. This was particularly a problem when using the CylinderMeasure tool; it is visually confusing when there are many cylinders (as produced by the measurement tool) present on the screen simultaneously. We need mechanisms for managing the display of these cylinders. Sometimes it is useful and important to be able see many at the same time, but sometimes it disrupts the user’s ability to understand the scene.

The clipping tools were useful in helping to alleviate some of the visual clutter and as an aid in isolating the elements of the scene to be measured. The BoxClip tool was far more useful than the WandClip tool because the box was not directly tied to the position of the wand. The clipping box could by placed and sized exactly as the user desired and remained stable during the measurement tasks. This was very effective in allowing the user to work on particular features of the data representations. This tool has been used quite productively in other applications, particularly in a project involving the visualization of a simulation of hydrating cement.

We also found that we needed mechanisms for grouping measurements. For example, the statistics that we show above have measurements grouped by layer or by orientation. This grouping was done in a post processing step. It would be very desirable to be able to specify the groupings and to show group statistics during the immersive session.

While 3D manipulations and interactions were greatly facilitated by the immersive environment, control functions such as specification of statistical analyses, saving disk files, and so on were hampered by the limitations of our interface. We were limited by the use of a simple pointing device and standard 2D GUI-based interfaces for such interactions. There were too many choices and options to effectively present the user with a full range of control.

Finally, it is worth considering the concept of presence and how it relates to our measurement tools. “Presence is defined as the subjective experience of being in one place or environment, even when one is physically situated in another.” [31] Presence is important to us only insofar as it furthers our goals of perceiving structure and spatial relationships and as it facilitates the use of our measurement tools. In the use of our tools, there is a strong sense of direct interaction with the 3D data representations. However breaks in the sense of presence are not uncommon, but neither are they troublesome. In any event, whether or not the user experiences a subjective sense of presence, we believe that the immersive visualization environment provides the ability to interact with virtual objects and tools much more effectively than would be possible with a desktop system.

## 8. Future Work and Conclusions

We are planning to continue to enhance and expand our set of measurement tools in a variety of ways. We plan to add tools that enable the interactive measurement of additional quantities. For example, we will need a tool for directly measuring angles (our current tools only allow this indirectly). We also see a need for tools that will measure the volume of regions of various shapes and tools for measurement of surface characteristics such as curvature and area. Our current tools need enhancements to improve their ease-of-use and accuracy. For example, we would like to have interactive dragging of objects or points that is geometrically constrained. For example, one might want to constrain the dragging of a point to a horizontal plane. While using the system we also often want the ability to place a point precisely onto an existing surface (a *snap-to* feature). We also wish to implement a mechanism for moving elements very small amounts without depending on having a very steady hand (a *tweak mode*). As described above, we also need mechanisms for hiding and revealing the visual items created by tools in the 3D scene in order to reduce visual clutter.

A very interesting future direction for this work will be the investigation of the interplay between manual measurement methods, as described here, and automatic measurement tools. For example, looking at the data for the Scaffold A samples, one could well imagine an algorithm for making an automatic fit of a cylinder to a strut, but will the automatic method produce satisfactory results? If we have automatic methods that produce mediocre results, we could implement a hybrid approach where the automatic methods produce preliminary measurements that are then interactively adjusted by the researcher in the immersive environment. The virtual environment could also be used to show the researcher the operation of automatic methods. For example, iterative methods could be displayed with time sequences of intermediate results.

One of the greatest problems to be addressed in this work revolves around user interface. As we give researchers more options for the analysis and display of new measurements in the immersive environment, we need to give them more ways of specifying those options. Standard 2D GUI techniques like menus, buttons, and sliders are useful, but they will not do the whole job. We need to provide users with the ability to specify a very wide range of choices with control of many parameters. This is a substantial challenge.

Because we have not performed formal user studies, we cannot definitively say that our measurement methods in the immersive environment are superior to equivalent methods that might be implemented on a desktop system. However we can say that these tools have been effective in making useful measurements of tissue engineering scaffold materials and that the immersive experience seems to have substantially contributed to the effectiveness of the tools. We look forward to performing usability studies in the future.

With these measurement tools and the means to analyze the resulting data during the interactive session, we are taking steps toward the implementation of a virtual laboratory. In this virtual laboratory, we can interact directly with data representations and acquire new quantitative insight into experiments and simulations.

## Figures and Tables

**Table 1:** Mean and standard deviation of measured vertical distances in μm between struts broken down by orientation group. Each orientation group is labeled according to its nominal orientation in degrees: 0, 60, or 120

	Strut	Syn Model		PCL1		PCL2	
	Groups	Mean	SD	Mean	SD	Mean	SD
Center to Center	0	1199.9	1.1	835.9	19.2	721.7	5.7
	60	1199.6	1.2	799.9	5.7	749.2	32.3
	120	1200.5	0.9	844.0	60.2	761.1	19.8
Edge to Edge	0	804.5	1.0	539.7	7.9	378.2	7.5
	60	801.1	0.9	473.6	10.9	399.4	27.2
	120	802.7	0.9	542.3	68.8	427.2	4.3

**Table 2:** Mean and standard deviation of measured angles in degrees between struts in different orientation groups

Strut Groups	Syn Model		PCL1		PCL2	
	Mean	SD	Mean	SD	Mean	SD
0 and 60	59.9	0.1	60.2	0.3	60.0	0.4
0 and 120	60.0	0.0	60.3	0.2	59.9	0.3
60 and 120	60.1	0.1	59.6	0.2	59.9	0.3

## Footnotes

1 Certain commercial equipment, instruments, or materials are identified in this paper to foster understanding. Such identification does not imply recommendation or endorsement by the National Institute of Standards and Technology, nor does it imply that the materials or equipment identified are necessarily the best available for the purpose.
